# The complete mitochondrial genome and phylogenetic analysis of *Cancer magister* (Decapoda, Cancridae)

**DOI:** 10.1080/23802359.2019.1691474

**Published:** 2019-11-20

**Authors:** Sigang Fan, Chao Zhao, Pengfei Wang, Lulu Yan, Lihua Qiu

**Affiliations:** a Key Laboratory of Aquatic Product Processing, Key Laboratory of South China Sea Fishery Resources Exploitation & Utilization, Ministry of Agriculture, South China Sea Fisheries Research Institute, Chinese Academy of Fishery Sciences, Guangzhou, PR China;; b Key Laboratory of Aquatic Genomics, Ministry of Agriculture, PR China

**Keywords:** *Cancer magister*, mitochondrial genome, phylogeny

## Abstract

The complete mitochondrial (mt) genome of *Cancer magister* was obtained using next-generation sequencing. The circular genome was 39,658 bp in length, consisting of 13 protein-coding genes, 26 transfer RNA genes, and 2 ribosomal RNA genes. Unfortunately, the control region was not found in mitochondrial genome. Of the 41 genes, 24 were encoded by the heavy strand, while the others were encoded by the light strand. The genome composition with A + T bias (69.90%). The phylogenetic analysis showed that *C. magister and Cancer pagurus* was clustered together, then grouped with *A. alayseae* and *Gandalfus puia*, which may suggest Cancroidea was close with Bythograeidae. The newly described mitochondrial genome may provide valuable data for phylogenetic analysis for Cancridae.

The Dungeness crab, *Cancer magister*, is a commercially and ecologically important crustacean that distributed along the eastern Pacific coast from Alaska to Santa Barbara (CA, USA) (Mclean and Todgham 2015). Females of *C. magister* carry eggs in the autumn. Approximately 90 d, females release planktonic larvae into the water column. After 2–3 years, *C. magister* reaches sexual maturity (Rasmuson 2013).*C. magister* is the most lucrative and valued at more than $200 million annually (Trigg et al. 2019).

Mitochondrial (mt) genome data have been widely used for phylogenetic, evolutionary studies, and population genetics in crabs (Ma et al. 2015, 2019). The mt genomes of crabs were usually represented by 37 genes, including 13 PCGs, large and small ribosomal RNA genes (rrn L and rrn S), 22 transfer RNA genes (tRNAs) (Karagozlu et al. 2018; Park et al. 2019). However, the availability of Dungeness crab mt genomes is limited. Therefore, the complete mt genome of *C. magister* and its phylogenetic relationships within crab were investigated in this study. The results of this study will provide essential information to genetic resources conservation and systematic study of *C. magister*.

Specimens of *Cancer pagurus* was collected from in Huangsha aquatic products market in Guangzhou (23°07′N, 113°5′8″E), Guangdong province, China and kept in the South China Sea Fisheries Institute (Guangzhou, China). Muscle was sampled and frozen in liquid nitrogen and stored at −80 °C. After sampling, the specimen was stored in 90% ethanol and deposited at the South China Sea Fisheries Research Institute Museum (Acc. Number CMGZ20190802). The mitochondrial DNA (mtDNA) was isolated by Mitochondrial DNA Isolation Kit (Haling Biotech Shanghai, Co., Ltd., Shanghai, China) and sequenced using the Illumina Hiseq Sequencing System (Illumina Inc., San Diego, CA). The clean data were acquired and assembled by the SPAdes and PRICE (Bankevich et al. 2012). The mitogenome was annotated by UGENE ORFs finder and tRNAscan-SE (http://www.cbs.dtu.dk/services/RNAmmer/).

In general, the complete mt genome of crab was 1.5–1.7 kb in length (Ma et al. 2019). However, the complete mitogenome sequence of *C. magister* was 39,658 bp in length (GenBank accession: MN371144), which was closer with *C. cancer* (Data were not open) and longer than other crabs. The overall base composition of *C. magister* mitogenome sequence is A-34.1%, T-35.8%, C-21.3%, and G-8.8%. The genome contained 13 protein-coding genes, 26 transfer RNA genes, and 2 ribosomal RNA genes. However, the control region was not predicted successfully in mitochondrial DNA sequence. Among 26 transfer RNA genes, there were 5 trnLs, 2 trnIs, 2 trnNs, and 2 trnAs, respectively. Twenty-six tRNA genes, ranged in size from 56 to 75 bp. Of the 41 genes, 24 were encoded by the heavy strand, and the others were encoded by the light strand. Nine protein-coding genes (*ND4*, *ND4L*, *ND5*, *ND6*, *ND2*, *COX2*, *COX1*, *ATP8*, and *COX3*) were initiated by ATG. ND1 and ATP6 were started by GTG. ND3 and Cytb were initiated by ATC and ATT, respectively. Eight PCGs (*ND6*, *ND2*, *ND1*, *ND4*, *ND4L*, *ND3*, *ATP6*, and *ATP6*) terminate with the typical TAA or TAG as a stop codon, while two PCGs (Cox1and Cox2) end with T––. *Cox3*, *DN5*, and *Cytb* ended with TA–,TT–, and TG–, respectively.

The phylogenetic tree was constructed based on 13 concatenated protein-coding genes from 22 crab species from Genbank database, by maximum likelihood (ML) method. *Harpiosquilla harpax* was used as an outgroup for tree rooting (Figure 1). It was demonstrated that *C. magister* and *C. pagurus* were clustered together, then grouped with *A. alayseae*, *G. puia*, and *M. planipes*, which may suggest Cancroidea was closely related with Bythograeidae and Matutidae. In all, this genome will contribute to future phylogenetic studies of Cancridae and population genetic analyses for *C. magister.*

**Figure 1.**
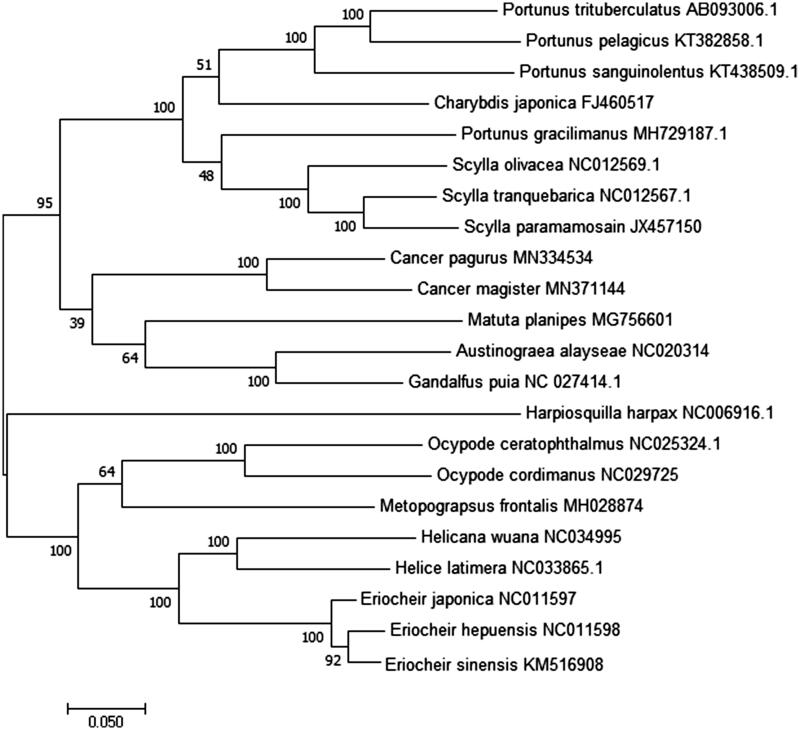
Phylogenetic tree of *C. magister* and related species based on maximum likelihood (ML) method with *Harpiosquilla harpax* as an outgroup.
